# MicroRNA-95 promotes myogenic differentiation by down-regulation of aminoacyl-tRNA synthase complex-interacting multifunctional protein 2

**DOI:** 10.18632/oncotarget.22796

**Published:** 2017-11-30

**Authors:** Biao Li, Shanshan Xie, Chunbo Cai, Lili Qian, Shengwang Jiang, Dezun Ma, Gaojun Xiao, Ting Gao, Jinzeng Yang, Wentao Cui

**Affiliations:** ^1^ Institute of Animal Sciences, Chinese Academy of Agricultural Sciences, Beijing, P. R. China; ^2^ Department of Human Nutrition, Food and Animal Sciences, University of Hawaii at Manoa, Honolulu, Hawaii, USA

**Keywords:** myostatin, miR-95, AIMP2, C2C12, differentiation

## Abstract

MicroRNA-95 (miR-95) is well known for its ability to promote the proliferation of a variety of cancer cells, but its function in skeletal muscle development has not been reported so far. Our laboratory has recently generated genetically engineered Meishan pigs containing a loss-of-function myostatin (MSTN) mutant (MSTN^-/-^). These MSTN^-/-^ pigs grow and develop normally but show clear double muscle phenotype as observed in Belgian cattle. We observed that the expression of miR-95 was up-regulated in the longissimus dorsi from MSTN^-/-^ Meishan pigs at day 65 during embryo development. In this study, we investigated the role of miR-95 in the myogenic differentiation using a murine myoblast cell line C2C12. Our results revealed that miR-95 may play a very important role in regulating the expression of myogenic differentiation marker genes myosin heavy chain (MHC) and myogenin. By use of bioinformatical analysis and luciferase reporter gene assay, aminoacyl-tRNA synthase complex-interacting multifunctional protein 2 (AIMP2) gene was identified as a miR-95 target gene involved in myogenic differentiation. Our results indicated that higher miR-95 expression level leads to lower level of AIMP2 protein expression. When the endogenous expression of AIMP2 is inhibited by siRNA, the expression levels of myogenic differentiation marker genes MHC and myogenin increased, implying that AIMP2 negatively regulates myogenic differentiation. Taken together, it is likely that miR-95 promotes myogenic differentiation in C2C12 myoblasts and may play a positive functional role in skeletal muscle development by down regulating the expression of AIMP2 at protein level.

## INTRODUCTION

As early as 1997, McPherron and Lee [1] identified a new TGF-β member called myostatin (MSTN) in mice. A series of *in vivo* studies such as gene knockout have confirmed MSTN’s inhibitory roles in muscle proliferation and development. Recently, gene editing methods such as nuclease-mediated zinc finger nucleases (ZFNs), transcriptional activator like effector nucleases (TALENs), and RNA-guided CRISPR-Cas nuclease (CRISPR/Cas9) have been widely used to make specific genetic modifications. Our lab has recently generated ZFN-edited MSTN loss-of-function mutant pigs that have the same apparent phenotype as the double muscle Belgian cattle [2]. These MSTN-edited Meishan pigs are as healthy as normal wild type pigs, but produce improved quality pork with greater lean yield and lower fat mass [2]. Muscle growth and development involves very complex regulatory processes. It has been demonstrated that skeletal muscle development is negatively impacted with incomplete hyperplasia in mice when endonuclease Dicer was conditionally knocked down [3–5], implying that microRNAs may play important roles in skeletal muscle development.

22 nt-length microRNAs (miRNAs) are a class of non-coding small RNAs that are widely found in plants and animals [6, 7]. Through the regulation of a variety of target genes, miRNAs are involved in cellular proliferation and differentiation [8, 9]. For example, miR-29, miR-181 and miR-148a can promote myoblast differentiation by inhibiting the expression of downstream target genes Akt3, Hox-A1 and ROCK1 at protein levels [10–12]. Chen et al. [13–15] previously reported that miR-1 and miR-206 can promote the differentiation of skeletal muscle satellite cells and significantly inhibit their proliferation by decreasing the expression level of Pax7. To better study the regulatory mechanism of MSTN in muscle development, we employed high-throughput sequencing technique to screen the differential expression of small RNAs in samples collected at day 65 during embryo development in wild type (WT) and MSTN-edited (MSTN^-/-^) Meishan pigs.

We observed that the expression level of miR-95 in skeletal muscle at day E65 was higher in MSTN^-/-^ than in WT Meishan pigs. However, most studies in the literature indicate that miR-95 is mainly associated with proliferation of cancer cells. Zhang et al. [16] reported that there is a significant increase in miR-95 expression in pancreatic cancer when compared with normal tissue. Huang et al [17] reported that miR-95 promoted the proliferation of colorectal cancer cells by directly down regulating Nexin 1 gene. Huang et al. [18] studied the relationship between miR-95 expression and radiation resistance and noted that miR-95 was directly involved in tumor’s resistance to radiation treatment by regulation of SGPP1. Chen et al [19] conducted a study on the role of miR-95 in promoting non-small cell lung cancer cell proliferation and identified SNX1 as the direct target gene. To our best knowledge, no study has been reported on the role of miR-95 in skeletal muscle cell development, cell cycle, and cell differentiation.

The expression product of aminoacyl-tRNA synthase complex-interacting multifunctional protein 2 (AIMP2) gene is a type 2 multifunctional protein that binds to human aminoacyl-tRNA synthase. AIMP2 gene is homologous to the glutathione S-transferase gene family, and thus AIMP2 protein contains the glutathione S-transferase (GST) domain, which binds to damaged DNA, thereby acting as a chaperone, resulting in the loss of reactive oxygen species, and ultimately promoting tumor cell apoptosis and mutagenesis [20–23]. Studies using AIMP2 knockout mice indicated that oncogene c-Myc can induce AIMP2 expression, implying thatAIMP2 may play a regulatory role in cell division cycle [23, 24]. AIMP2 may be a multifunctional protein that may be widely involved in many basic cellular processes such as inflammation, cell division, cell differentiation, aging, apoptosis, death and tumorigenesis. It has been reported that, through the regulation of TGF-β, AIMP2 protein is translocated into the nucleus and then interacts with FUSE-binding protein (FBP) and down-regulates the expression of c-Myc and the subsequence inhibition of proliferation [25]. There are few reports on the relationship between AIMP2 gene expression and muscle development, so our current study focused on this issue.

Myogenin and myosin heavy chain (MHC) are typical markers genes of cell differentiation. Myogenin is well known to regulate terminal differentiation, it is expressed during differentiation. Cells enter myogenic differentiation phase following the expression of myogenin, which subsequently results in the expression of MHC, indicating that the differentiation of myogenic differentiation into the late stage. Therefore, detection and measurement of myogenin and MHC are very important to monitor muscle cell differentiation.

Here in this study, we investigated the regulatory role of miR-95 in skeletal muscle development by use of bioinformatical analysis and MSTN^-/-^ Meishan pigs as a model. We observed that miR-95 expression was up-regulated in the longissimus dorsi from MSTN^-/-^ Meishan pigs at day 65 during embryo development. The role of miR-95 in myogenic differentiation was further investigated using a murine myoblast cell line C2C12. We identified AIMP2 as the direct target gene for miR-95. Our results demonstrated that there is an inverse relationship between the expression level of miR-95 and AIMP2 protein level: higher expression level of porcine miR-95 reduced AIMP2 protein level, while the inhibition of miR-95 increased AIMP2 protein level. Additionally, there is a positive relationship between miR-95 expression and the expression level of myogenic differentiation marker genes MHC and myogenin. By use of bioinformatical analysis and luciferase reporter gene assay, AIMP2 gene is identified as a miR-95 target gene involved in myogenic differentiation. When the endogenous expression of AIMP2 is inhibited by siRNA, the expression levels of myogenic differentiation marker genes increased, implying that AIMP2 negatively regulates myogenic differentiation. Taken together, our results demonstrated that miR-95 promotes myogenic differentiation in C2C12 myoblasts and thus may play a positive functional role in regulating muscle development by specifically down regulating the expression of AIMP2 at protein level.

## RESULTS

### miR-95 is up-regulated in MSTN^-/-^ meishan pigs at day E65

**Figure 1:**
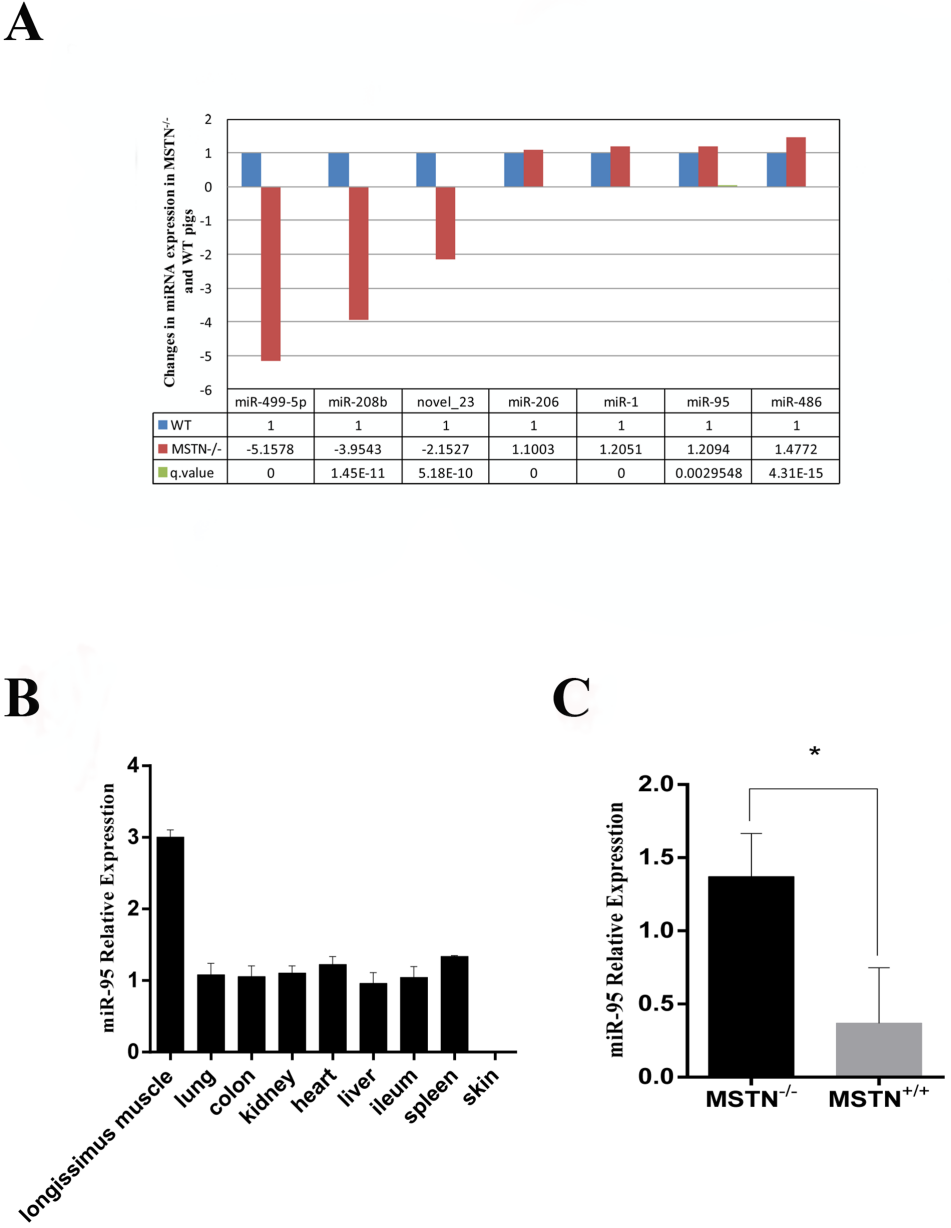
Up-regulation of miR-95 in MSTN^-/-^ Meishan pig at day E65 **(A)** Changes in expression levels of several microRNAs in muscles of MSTN^-/-^ and WT pigs on day E65. Left panel represents the cluster analysis of differentially expressed siRNAs. **(B)** RNA was extracted from different tissues of WT Meishan pigs on day E65, and the expression of miR-95 was detected by q-RT-PCR. **(C)** RNA was extracted from muscles of MSTN^-/-^ and WT pigs on day E65 and miR-95 expression level was determined by q-RT-PCR (mean ± SEM, n = 3 pigs, ^*^P<0.05).

miRNA deep sequencing was performed for skeletal muscle samples collected at day E65 from MSTN^-/-^ and WT Meishan pigs. We have identified several miRNAs that are up-regulated in MSTN^-/-^ pigs, and these miRNAs have previously been shown to be involved in myoblast development, including the well-known miR-1, miR-206 [13, 15], and miR-486 [26] (Figure 1A). Analysis of miR-95 expression levels in different tissues from WT pigs indicated that miR-95 is highly expressed in skeletal muscle compared to other tissues (Figure 1B) at day E65. We then further measured miR-95 expression level from three individual WT and MSTN^-/-^ pigs, respectively, by using the TagMan-miRNA Expression Assay and confirmed that miR-95 expression in skeletal muscle is higher in MSTN^-/-^ pigs than in WT pigs (Figure 1C).

### miR-95 promotes C2C12 cell cycle arrest

**Figure 2:**
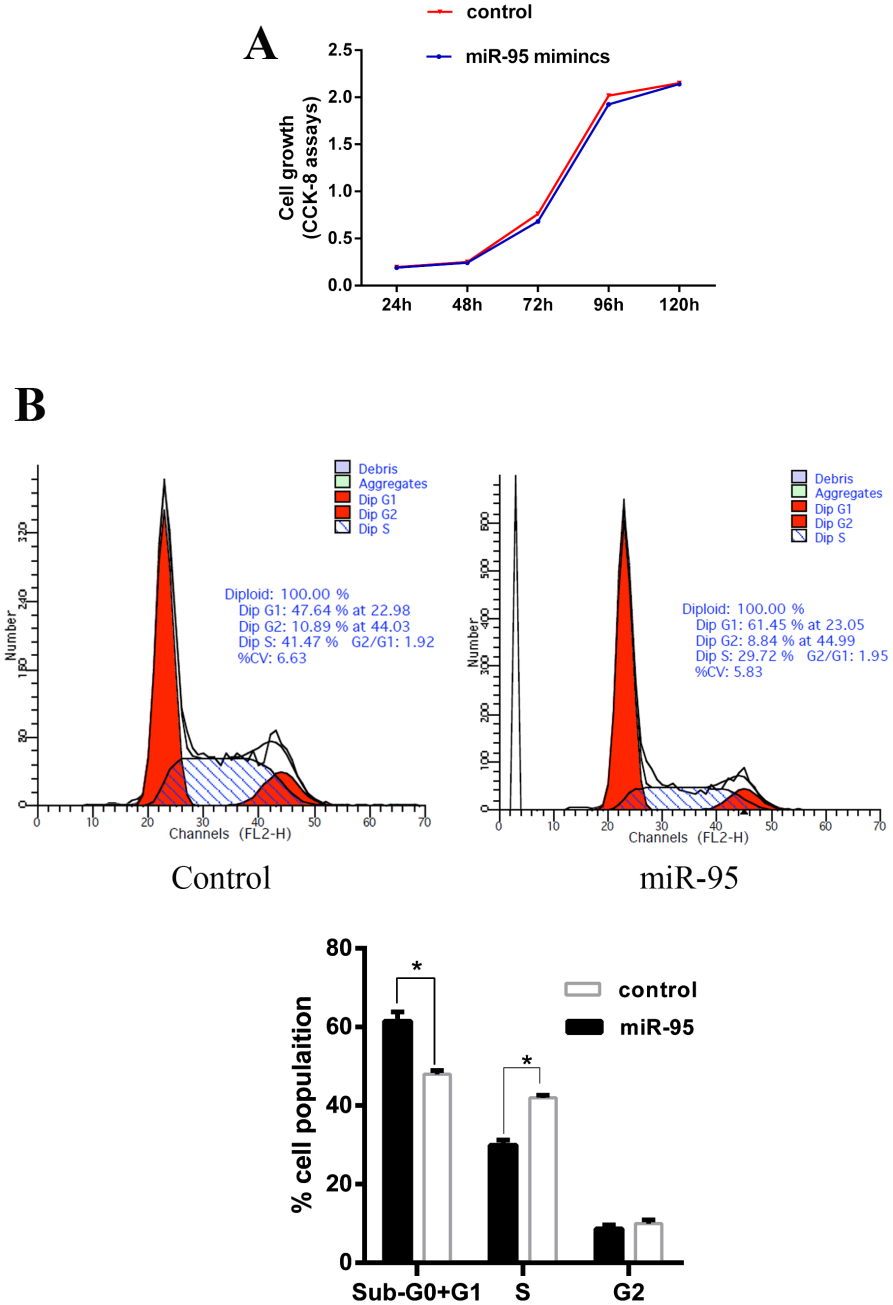
Effect of miR-95 mimics on C2C12 cell proliferation and cell cycle **(A)** Growth curve of C2C12 myoblasts transfected with either the miR-95 mimic or negative control. Data was collected at 24, 48, 72, 96, and 120 hours. Each value represents the average of three measurements. **(B)** Cells were harvested at 36 hours and cell cycle analysis was performed using flow cytometry to detect the proportion of cells at G1 phase, S phase, and G2 phase (^*^P<0.05).

It was speculated that based on the fact that the expression of miR-95 in skeletal muscle is higher in MSTN^-/-^ than in WT Meishan pigs at day E65. To investigate the role of miR-95 in skeletal muscle development, murine myoblast cell line C2C12 was transfected with porcine miR-95. We confirmed that the expression of porcine miRNA-95 in transfected C2C12 cells is 60 times higher compared to control (non-transfected) cells (data not shown). We observed that the high expression level of porcine miR-95 had no significant effect on C2C12 cell proliferation (Figure 2A). Flow cytometry analysis indicates that, compared to control cells, C2C12 cells transfected with porcine miR-95 contained significantly greater proportion of cells at G1 phase and significantly lower proportion of cells at S phase (Figure 2B), suggesting that the high expression level of porcine miR-95 led to a cell cycle arrest in C2C12 cells. This result suggests that miR-95 may play an important role in regulating the differentiation of C2C12 cells.

### Increased expression of miR-95 during C2C12 cell myogenic differentiation

**Figure 3:**
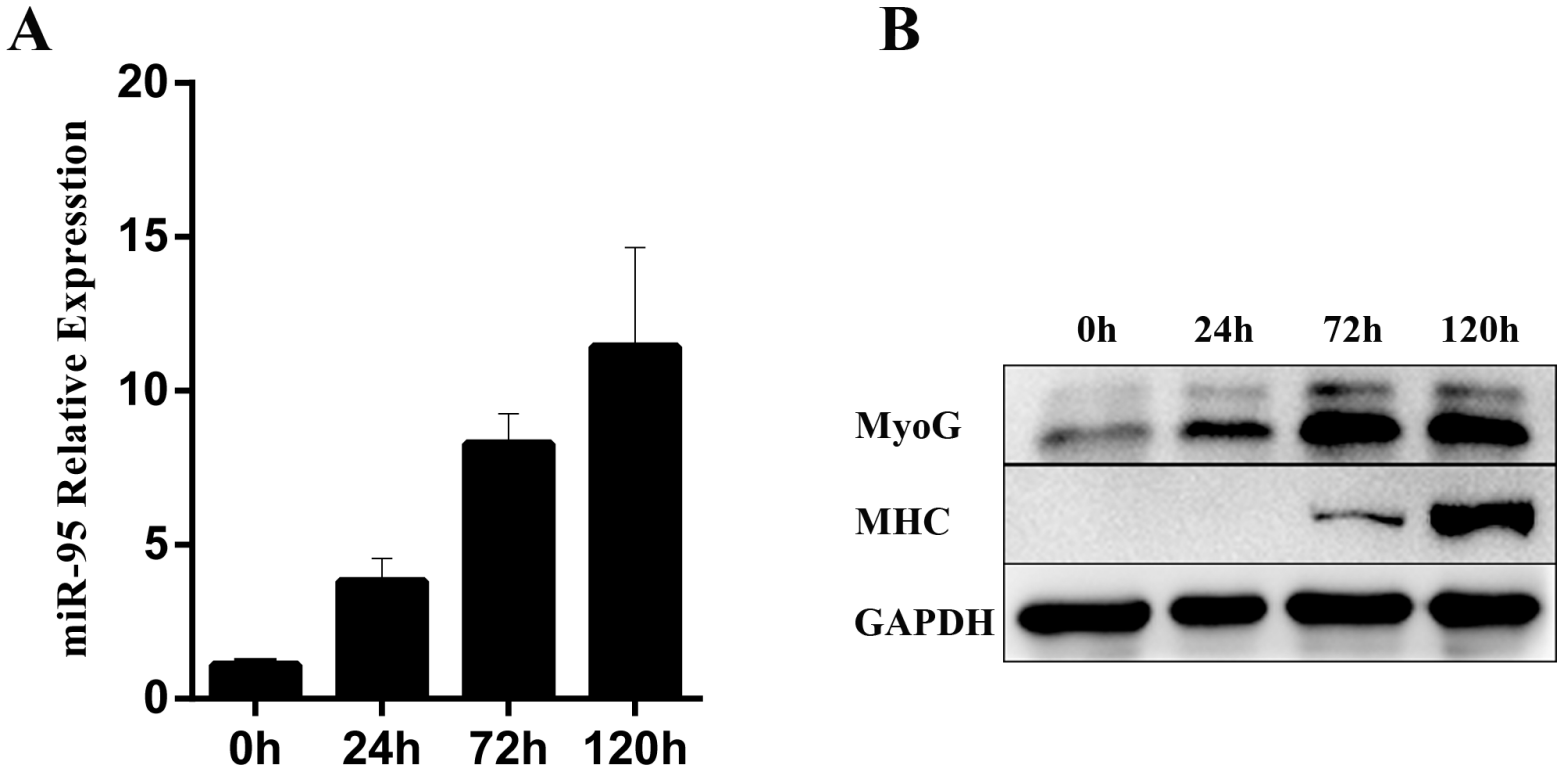
Changes in expression levels of miR-95 and differentiation marker genes MHC and myogenin in C2C12 cells during *in vitro* differentiation **(A)** The expression of miR-95 was detected by TaqMan qPCR at 0, 24, 72 and 96 hours. **(B)** The protein expression of differentiation marker genes MHC and myogenin was detected by Western blot.

We further examined the expression pattern of miR-95 during C2C12 cell myogenic differentiation. Myogenic differentiation of C2C12 cells was first induced in by replacing 10% fetal bovine serum with 2% equine serum in the cell culture medium. During the differentiation process from 0 to 120 hours, we observed that the expression of miR-95 increased gradually (Figure 3A). At the same time, the expression of two differentiation marker genes, MHC and myogenin, also increased significantly during the differentiation process, indicating that both miR-95 and the two differentiation marker genes follow the same trend during C2C12 cellular differentiation process (Figure 3B).

### miR-95 positively regulates myogenic differentiation

**Figure 4:**
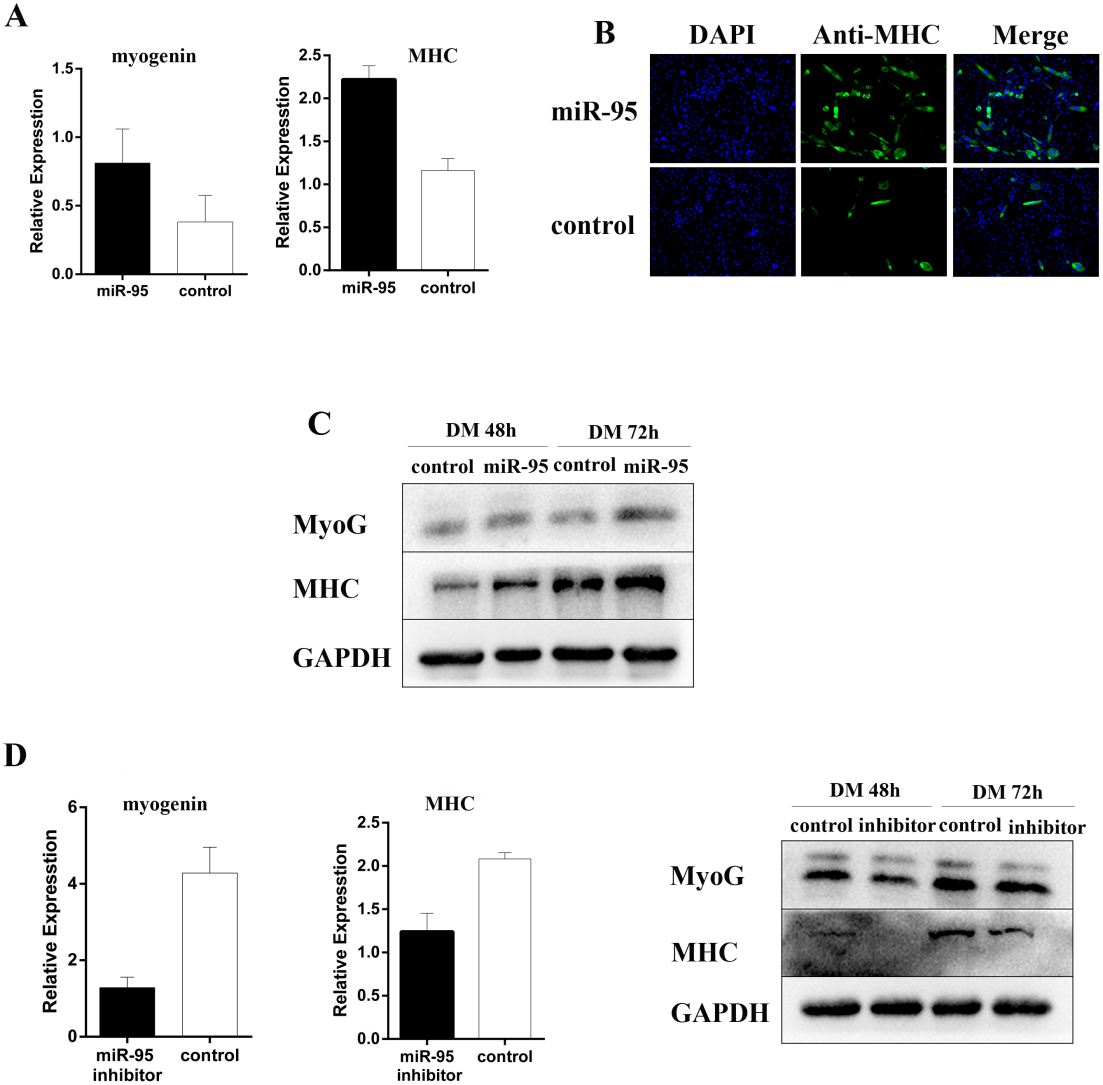
Positive regulation of myoblast differentiation by miR-95 **(A)** Expression of MHC and myogenin mRNA detected by qPCR by at 48 hours of differentiation. **(B)** The number of MHC positive cells was analyzed by immunofluorescence at day 3 after the induction of differentiation. **(C)** The expression of myogenin and MHC detected by Western blot at 48 and 72 hours following differentiation. **(D)** The expression of myogenin and MHC mRNA detected by qPCR (left and middle panels) or protein detected by Western blot (right panel) at 48 hours following differentiation.

To examine the function of miR-95 in myoblast differentiation, we designed and synthesized a double-stranded mimic for porcine miR-95 RNA and a single-stranded inhibitor against porcine miR-95, and then transfected C2C12 myoblast cells with each vector, respectively. As shown in Figure 4A, miR-95 significantly enhanced the expression levels of differentiation marker genes MHC and myogenin. Immunofluorescence assay showed that miR-95 significantly increased the total number of MHC-positive cells (Figure 4B). Immunoblotting revealed that overexpression of miR-95 increased MHC and myogenin expression at protein level (Figure 4C). On the other hand, the expression of myogenic differentiation marker genes MHC and myogenin decreased significantly at both mRNA and protein levels in C2C12 cells transfected with a vector containing the miR-95 inhibitor (Figure 4D). Taken together, these results clearly demonstrated that the expression level of miR-95 is very closely associated with the expression levels of differentiation marker genes and subsequently with myogenic differentiation in C2C12 cells.

### Effect of miR-95 on AIMP2 protein expression during myogenic differentiation

**Figure 5:**
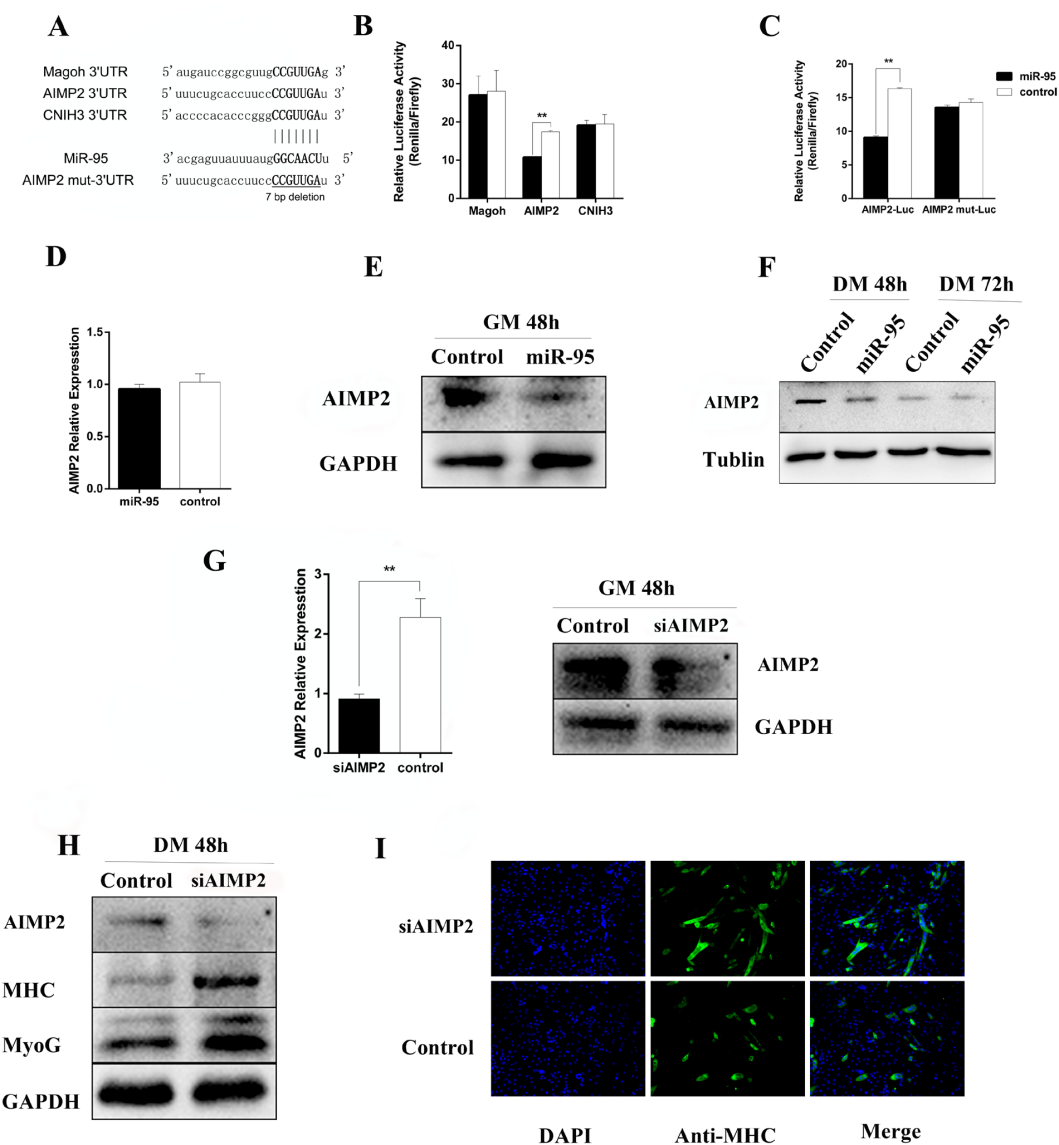
Regulation of AIMP2 at protein level by miR-95 **(A)** Prediction of the miR-95 binding sites to3'UTR in murine Magoh, AIMP2, and CNIH3. The base in the seed region (bold line) of AIMP2 mut 3'UTR has been deleted. **(B)** Luciferase activity of 2031T cells at 36 hours following transfection with miR-95 mimic or negative control along with luciferase vectors containing 3'UTR of Magoh, AIMP2, and CNIH3, respectively. **(C)** Luciferase activity of 2031T cells at 36 hours following transfection with miR-95 mimic or negative control along with luciferase vectors containing AIMP23'UTR or 3'UTR mutant, respectively (^**^p < 0.01). **(D)** mRNA level of AIMP2 (detected by qPCR) in C2C12 cells transfected with miR-95 mimic or negative control at 48 hours. **(E)** Protein expression level of AIMP2 (detected by Western blot) in C2C12 cells transfected with miR-95mimics or negative control at 48 hours. **(F)** Time course of protein expression of AIMP2 (detected by Western blot) during the growth of C2C12 cells transfected with miR-95 mimic or negative control at 48- and 72- hours. **(G)** Protein expression level of AIMP2 (detected by Western blot) during growth of C2C12 cells transfected with miR-95 inhibitor or negative control at 48 hours. **(H)** Expression level of AIMP2 (detected by qPCR and Western blot) in C2C12 cells transfected with AIMP2 siRNA or negative control at 24 and 48 hours. **(I)** Expression level of AIMP2, MHC, and myogenin detected by Western blot inC2C12 cells transfected with AIMP siRNA or negative control at 48 hours post differentiation induction. **(J)** Detection of MHC-positive cells by immunofluorescence analysis in C2C12 cells transfected with AIMP siRNA or negative control at 72 hours.

**Figure 6:**
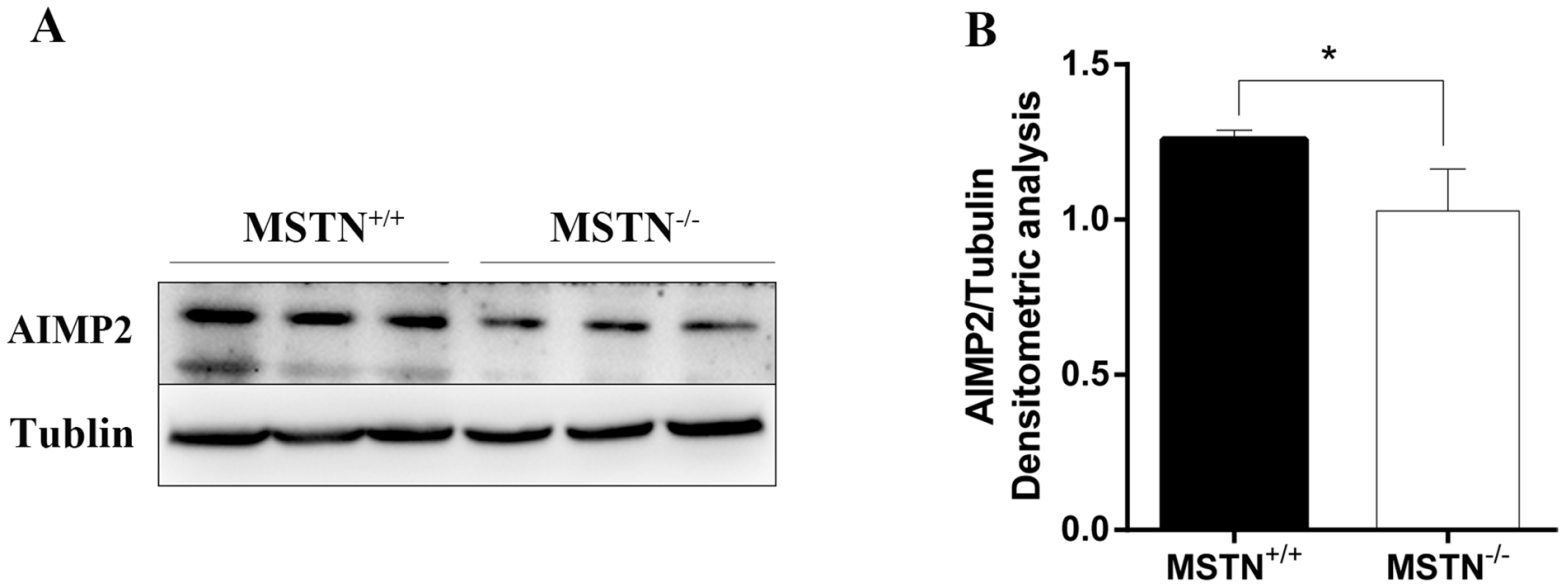
AIMP2 expression level in longissimus dorsi **(A)** Samples were collected at day E65 from both wild type (MSTN^+/+^) and MSTN-edited (MSTN^-/-^) Meishan pigs, and AIMP2 level was as detected by Western blot. **(B)** Normalized AIMP2 protein level relative to Tublin as determined by band intensity (mean ± SEM, n = 3 pigs, ^*^p<0.05).

By using software analysis with RNAhybird, PITA, and Miranda to predict the relevant target genes, we identified three genes as potential targets for mi-R95. After sequence comparison, it was noted that the 3 'untranslated region (UTR) of Magoh, AIMP2, and CNIH3 mRNA sequences are complementary to porcine miR-95 seed sequence (Figure 5A). To verify if Magoh, AIMP2, and CNIH3 are direct targets for miR-95, we cloned 3'UTR of each target gene into the downstream of luciferase ORF in psiCHECK2 vector, and then co-transfected HEK-293T cells with miR-95. It was noted that miR-95 significantly down-regulates the luciferase activity of AIMP2 3'UTR (Figure 5B), with significant effect on 3'UTR of Magoh and CNIH3. We further designed the AIMP2 3'UTR mutants (AIMP2 3'UTR sequence is: 5’CCAAGCTGCACTACAAGAGAACTCCTTGACAAACATTTTTAAAGGTCATGGAACAACCATAACCTTCCCCATTGATTATTAAGGTCCTTTCTGCACCTTCCCCGTTGATTATTAAGGTCCTTTTTGCACCTTCCCCGTTGATTATTGAGGTCCTTTTTGCACCTTCCCCGTTGATTATTAAGGTCCTTTTTGCACCTTCCCCATTGATT3’, and AIMP2 mut-3'UTR sequence is: 5’CCAAGCTGCACTACAAGAGAACTCCTTGACAAACATTTTTAAAGGTCATGGAACAACCATAACCTTCCCCATTGATTATTAAGGTCCTTTCTGCACCTTCCTTATTAAGGTCCTTTTTGCACCTTCCCCGTTGATTATTGAGGTCCTTTTTGCACCTTCCCCGTTGATTATTAAGGTCCTTTTTGCACCTTCCCCATTGATT3’) and co-transfected HEK-293T cells with miR-95 and AIMP2 3'UTR mutant. No significant change in luciferase activity was observed (Figure 5C) for AIMP2 3'UTR mutant, indicating that miR-95 specifically targeting AIMP2 3'UTR. In addition, we analyzed the level of AIMP2 protein by Western blotting in samples collected at day E65. The Western blot results revealed that the expression level of AIMP2 protein at day E65 in the MSTN^-/-^ Meishan pigs (where miR-95 expression level is high) is lower than in WT Meishan pigs(where miR-95 expression level is low) (Figure 6), clearly demonstrating that miR-95 negatively regulates AIMP2 expression at protein level.

To further explore how porcine miR-95 regulates the expression level of endogenous AIMP2 in C2C12 cells, C2C12 cells were transfected with porcine miR-95. AIMP2 expression at both mRNA and protein levels was then examined 24 hours and 48 hours following C2C12 cell transfection with miR-95 vector. Although RT-qPCR showed no significant decrease in AIMP2 mRNA expression (Figure 5D), Western blot result revealed a significant decrease in level of AIMP2 protein (Figure 5E). Furthermore, Western blot results revealed that AIMP2 protein level decreased during the course of C2C12 differentiation (Figure 5F). Although the inhibition of miR-95 expression up-regulated AIMP2 expression, the increased level of AIMP2 is not statistically significant (data not shown).

Endogenous AIMP2 expression was knocked down in C2C12 cells by AIMP2 siRNA to further study the relationship between AIMP2 and differentiation marker genes, MHC and myogenin (Figure 5G). The expression of MHC and myogenin protein in C2C12 myoblasts were significantly up-regulated when endogenous AIMP2 expression level is reduced (Figure 5H). Result from cell immunofluorescence staining showed that the number of MHC-positive cells increased significantly following knock down of AIMP2 (Figure 5I), demonstrating that AIMP2 plays a negative regulatory role during skeletal muscle differentiation.

## DISCUSSION

The formation and development of muscle probably includes the following three stages: myoblast differentiation, myoblast migration, and proliferation, and the formation of multinucleated myoblast/myotubes. However, the growth and development of muscle tissue is a complex regulatory process, and muscle precursor cells grow and develop into muscle under the fine regulation of a variety of factors [27–31]. Recent studies have revealed that non-coding RNAs (miRNAs) play crucial roles in skeletal muscle development. In this study, for the first time, we reported that miR-95 was up-regulated during the differentiation process of C2C12 myoblasts. Higher expression level of porcine miR-95 in C2C12 cells resulted in a significant increase in myogenic differentiation marker genes MHC and myogenin at both mRNA and protein levels. On the other hand, inhibition of miR-95 expression in C2C12 cells led to a decrease in myogenic differentiation marker genes MHC and myogenin at both mRNA and protein levels. These data indicate that miR-95 may play a positive role in regulating muscle differentiation.

Compared with the control group, the proportion of cells at G1 phase and at S phase was significantly higher and lower, respectively, in C2C12 cells transfected with porcine miR-95 (p <0.05). It has been known that arrest of cells at G1 phase is a critical step during differentiation. A previous study showed that miR-322/424 and miR-503 can promote cell differentiation by enhancing cell cycle arrest through the inhibition of cell cycle regulator Cdc25A [32]. MiR-148a, miR-206 and miR-214 have been shown to be similar to miR-322/424 and miR-503 [12, 33, 34]. Our results demonstrate that higher expression level of porcine miR-95 can arrest cells at G1 phase during the cell cycle and thus facilitates cells into differentiation stage. However, it is not clear what is the underlying mechanism in this process and further studies are required in the future. Although miR-95 can arrest cell cycle at G1 phase, there is no impact on the growth of C2C12 cells by either higher expression level of porcine miR-95 or inhibition of miR-95. This is a dramatic difference from what has been observed in cancer cells, where miR-95 promotes cell proliferation. It is suspected that there may be functional differences of miR-95 in different types of cells or tissues.

It is well known that most miRNAs function by regulating the expression levels of downstream target genes [9, 35]. One miRNA could simultaneously regulate the expression of multiple target genes. Since each target gene may play a role in different specific signaling pathway, and thus these target genes can form a complex and sophisticated regulatory network that will eventually amplify the function of each individual miRNA. In the luciferase reporter assay, we confirmed that AIMP2 is the target gene for miR-95 by transfecting C1C12 cells with vectors containing either miR-95 mimic or mutant. However, we cannot determine if miR-95 inhibits mRNA gene expression by mRNA degradation or by inhibition at the translation step. To further verify the mechanism by which miR-95 regulates the expression of endogenous AIMP2, we transfected porcine miR-95 in C2C12 myoblasts, and then measured changes in mRNA and protein levels of endogenous AIMP2 by qPCR and Western blot. Our results showed that the high expression level of porcine miR-95 did not affect the expression of endogenous AIMP2 at mRNA level, but rather it inhibited AIMP2 expression at protein level (see Figure 5D-5E), indicating that the inhibition of AIMP2 protein by high level of miR-95 expression is by blocking protein translation. We also observed that although the inhibition of miR-95 expression up-regulated AIMP2 expression as expected, the increased level of AIMP2 expression is not statistically significant. Since one miRNA can regulate multiple target genes, and at the same time, one single target gene can also be regulated by serval miRNAs, therefore, it is highly possible that the reason for miRNA-95 inhibition not significantly up-regulating AIMP2 at protein level is due to the fact that other miRNAs or other factors may be involved in AIMP2 regulation when these C2C12 cells were incubated in growth culture medium.

MSTN inhibits muscle development by activating different signaling pathways, for example, it negatively regulates myoblast differentiation by the inhibition of IGF-2 expression in the ALK-Smad signaling pathway [36]. Additionally, many other transcription factors such as FOXO were found to be involved in the regulation of MSTN [37, 38]. Although MSTN can suppress muscleigenesis by being involved in intracellular complex signaling pathways and by regulating the expression of target genes [39, 40], the potential underlying molecular mechanisms are yet to be fully understood. Along this line, we found that there are significant differences in the miRNA expression levels between MSTN^-/-^ Meishan pigs and WT pigs at day E65. This finding led to the identification of miR-95as a potential molecule involved in myogenic differentiation. We further observed that AIMP2 protein level in day E65 longissimus dorsi samples is significantly inhibited in MSTN^-/-^ Meishan pigs when compared to WT pigs. Although there are so many reports on the roles of miR-95 in cancer cell growth, our current study is the first to demonstrate a regulatory role of miR-95 in myogenic development, and identified AIMP2 as a target gene for miR-95. AIMP2 is an important scaffold protein in the cytoplasm and it can be recruited by AKT/PKB upstream kinase PDK-1. AIMP2 has been shown to inhibit the activation of AKT pathway by inhibiting the kinase activity of PDK-1 [22, 41]. The activation of AKT pathway plays an important role in many biological processes such as cell metabolism, cell survival, cell cycle regulation and other activities. Our lab [42] recently reported that the degree of AKT phosphorylation in skeletal muscle is significantly greater in MSTN^-/-^ Meishan pigs than in WT pigs, but further studies are needed to understand if there is any relationship between MSTN and miRNA. Hitachi et [43] reported that the expression level of miR-486 in skeletal muscle was significantly increased, and showed that, miR-486, which is a positive regulator of the IGF-1/Akt pathway, is involved in myostatin signaling in myostatin knockout mice. It has been reported that MSTN not only inhibits myoblast proliferation and differentiation, but also reduces the expression levels of differentiation marker genes myogenin and MHC in skeletal muscle [40]. Therefore, our results in this study using C2C12 cells provide a potential link between miR-95 mediated myogenic differentiation in C2C12 cells and the phenotype in MSTN^-/-^ pigs.

## MATERIALS AND METHODS

### Cell culture

C2C12 myoblast cells were purchased from the Cell Resource Center in IBMS in CAMS/PUMC. C2C12 myoblasts were cultured in Dulbecco’s modified Eagle’s medium (DMEM, Invitrogen) supplemented with 10% FBS (Hyclone) and 1% penicillin/streptomycin (Invitrogen). Myogenic differentiation was induced by replacing the medium of the subconfluent cells with DMEM medium supplemented with 2% horse serum (Hyclone) and 1% penicillin/streptomycin.

### RNA isolation

Total RNA was extracted using the TRI-zol reagent (Invitrogen) per manufacturer’s recommendations. RNA preparations with an A260/A280 ratio of 1.8–2.0 and an A260/A230 ratio greater than 2.0 were used for subsequent analysis.

### Immunoblotting and immunofluorescence

Immunoblotting was performed using standard procedures and antibodies against MHC (MF20, DSHB), myogenin (F5D, Abcam), AIMP2 (AIMP2/P38, Abcam), α-tubulin (Cell Signaling Technology), and GAPDH (Abcam). For immunostaining, C2C12 cells in 6-well plates were fixed in 4% formaldehyde for 10 min and then washed with PBS three times, 10 min each wash. The cells were then permeabilized with 0.1% Triton X-100 for 10 min. After blocking with 5% skim milk in PBS, the cells were incubated with the primary antibody MF20 (1:40 dilution) for 1 h at 37 °C. The Cy-3-conjugated anti-mouse IgG (1: 400 dilution) was incubated for 1 h at 37 °C. The nuclei of the cells were visualized using DAPI staining.

### RNA oligonucleotides and transfection

The miRNA mimics (double-stranded RNA oligonucleotides) and negative control duplexes were synthesized by Sangon Biotech. 2-O-methyl antisense oligonucleotides against the target miRNAs and a negative control were synthesized by Invitrogen. The miR-95 inhibitor sequence is: 5’-UGCUCAAUAAAUACCCGUUGAA-3’. Transfection was performed with the Lipofectamine 2000 reagent (Invitrogen) combined with 100 nM of miRNA mimics and 200 nM 2-O-methyl antisense oligonucleotide.

### TagMan^®^ miRNA expression assays

Single-stranded cDNA was synthesized from total RNA samples using specific miRNA stem-loop primers and the TaqMan^®^ MicroRNA Reverse Transcription Kit (Applied Biosystems). Mature miRNA expression was measured with TagMan^®^ MicroRNA Assays (Applied Biosystems) according to the manufacturer’s instructions with the Applied Biosystems 7500 Real-Time PCR system.U6 was used to normalize miRNA expression.

### Cell proliferation assay

C2C12 cells transfected with the miR-95 mimics or the negative control duplexes were seeded at 3×10^3^ cells/well in a 48-well plate and cultured in growth medium for 5 days. The cell proliferation assay was performed by adding 20 μL of Cell-Counting Kit-8 (CCK-8) reagents (Dojindo) to the cells and let them grow for 1 h. Absorbance at 450 nm was measured using the SpectraMax M5 microplate spectrophotometer.

### Flow cytometry analysis of the cell cycle

C2C12 cells were collected 36 h after transfection with either miR-95 mimics or the negative control duplexes, followed by washing with PBS and fixing in 75% ethanol at -20°C. For cell cycle analysis, 2-5×10^5^ cells from each sample were stained with 50μg/mL propidium iodide (Invitrogen) containing 10 μg/mL RNaseA (Takara) and then analyzed in a FACSCalibur flow cytometer (BD Biosciences).

### Quantitative real-time PCR for microRNA and mRNA quantification

Total RNA was extracted from C2C12 cells with the TRI-zol Reagent (Invitrogen). Each sample (1 μg) was reverse-transcribed into cDNA by using the RevertAid™ First Strand cDNA Synthesis Kit (Fermentas). Real-time PCR was performed in the Applied Biosystems 7500 Realtime PCR system using SYBR Premix ExTagTM (Takara) according to the manufacturer’s protocols. The housekeeping gene actin was used as an internal normalization control. The primers used for RT-qPCR are listed in Supplementary Table 1.

### Plasmids construction

The region of AIMP2 3’-UTR flanking the miR-95 binding site was amplified from mouse genomic DNA using PCR and the following specific primers: 5’-AACCTGCATGTACCGGCTC-3’ and 5’-CAAGGGCTTGCAAAGAAGGC-3’. The PCR product was cloned into the downstream of the Renilla Luciferase ORF (Promega, Madison, WI) using the NotI and XhoI restriction sites of vector psiCHECK-2. A psiCHECK-2 luciferase reporter with a mutant 3’-UTR of AIMP2 that has a 7 base pair (bp) deletion in the target sites was obtained from Shanghai Sangon Biotech.

### Dual luciferase reporter assay

293T cells were co-transfected with 100 ng of the wide-type or mutant 3’UTRluciferase reporter and 40 nM of the miR-95 mimics or the negative control duplexes using the Lipofectamine 2000 reagent (Invitrogen) in 24-well plates. Forty hours post transfection, the cells were harvested by adding 300uL of a passive lysis buffer. Renilla and firefly luciferase activities were measured with the Dual Luciferase Assay System (Promega, Madison, WI) in a TD-20/20 luminometer (Turner Biosystems, Sunnyvale, CA), and the Renilla luciferase signal was normalized to the firefly luciferase signal. The normalized Renilla luciferase activity was compared for miR-95 mimics, mutant, and negative control using the Student’s t test (p<0.05).

### Small interfering RNA (siRNA) against AIMP2 and transfection

The mouse AIMP2 ON-TRAGET plus SMARTpoolsiRNA was from Dharmacon. A nonspecific duplex was used as the control. Transfection was performed with the Lipofectamine 2000 reagent (Invitrogen) combined with 100nM of anti-AIMP2 siRNA.

### Target analysis

Bioinformatical analysis was performed by using these specific programs: RNAhybird, PITA and Miranda. Three pieces of program predict that 3′ UTRs of Magoh, AIMP2, and CNIH3 mRNA have miR-95 binding sites.

### Data analysis

All data were analyzed by using unpaired 2-tailed Student's t tests; p< 0.05 (^*^), p< 0.01 (^**^). Data were expressed as mean ± S.E.M (standard error of mean). Investigators were not blinded during allocation of experiment groups or during result analysis.

## SUPPLEMENTARY MATERIALS TABLE



## Abbreviations

MSTN: Myostatin
ZFNs: Zinc-fingers Nuclease
TALEN: Transcription Activator-Like Effector Nuclease
Akt: Serine/threonine kinase
Hox-A1: homeobox-A1
ROCK1: Rho-associated coiled coil-containing protein kinase 1
HDAC4: Histone deacetylase 4
PTEN: phosphatase and tensin homologue deleted on chromosome ten
Foxo1: Forkhead box protein O1
SNX1: Sorting Nexin 1
SGPP1: sphingosine-1-phosphate phosphatase 1
CUGBP2: CUG triplet repeat, RNA binding protein 2
AIMP2: aminoacyl-tRNA synthetase-interacting multifunctional protein type 2
p53: Tumor protein p53
MDM2: Mouse double minute 2 homolog
c-Myc: oncogene protein c-myc
FBP: FUSE-binding protein
TGF-β: Transforming growth factor beta superfamily
RT-PCR: Real time PCRMHC
Magoh: the human homolog of Drosophila magonashi
CINH3: cornichon family AMPA receptor auxiliary protein 3
IGF-2: insulin like growth factor 2
FOXO: Fraternal Order of X-Offenders
PDK-1: purinenucleotid-dependent-protein
FBS: Fetal bovine serum
DMEM/F12: Dulbecco's Modified Eagle Media: Nutrient Mixture F-12

## References

[1] McPherron AC, Lawler AM, Lee SJ (1997). Regulation of skeletal muscle mass in mice by a new TGF-beta superfamily member. Nature.

[2] Qian L, Tang M, Yang J, Wang Q, Cai C, Jiang S, Li H, Jiang K, Gao P, Ma D, Chen Y, An X, Li K, Cui W (2015). Targeted mutations in myostatin by zinc-finger nucleases result in double-muscled phenotype in Meishan pigs. Sci Rep.

[3] Williams AH, Liu N, van Rooij E, Olson EN (2009). MicroRNA control of muscle development and disease. Curr Opin Cell Biol.

[4] Callis TE, Chen JF, Wang DZ (2007). MicroRNAs in skeletal and cardiac muscle development. DNA Cell Biol.

[5] Bernstein E, Kim SY, Carmell MA, Murchison EP, Alcorn H, Li MZ, Mills AA, Elledge SJ, Anderson KV, Hannon GJ (2003). Dicer is essential for mouse development. Nat Genet.

[6] Ambros V (2004). The functions of animal microRNAs. Nature.

[7] Bartel DP (2004). MicroRNAs: genomics, biogenesis, mechanism, and function. Cell.

[8] Zeng Y, Yi R, Cullen BR (2003). MicroRNAs and small interfering RNAs can inhibit mRNA expression by similar mechanisms. Proc Natl Acad Sci U S A.

[9] Friedman RC, Farh KK, Burge CB, Bartel DP (2009). Most mammalian mRNAs are conserved targets of microRNAs. Genome Res.

[10] Wei W, He HB, Zhang WY, Zhang HX, Bai JB, Liu HZ, Cao JH, Chang KC, Li XY, Zhao SH (2013). miR-29 targets Akt3 to reduce proliferation and facilitate differentiation of myoblasts in skeletal muscle development. Cell Death Dis.

[11] Naguibneva I, Ameyar-Zazoua M, Polesskaya A, Ait-Si-Ali S, Groisman R, Souidi M, Cuvellier S, Harel-Bellan A (2006). The microRNA miR-181 targets the homeobox protein Hox-A11 during mammalian myoblast differentiation. Nat Cell Biol.

[12] Zhang J, Ying ZZ, Tang ZL, Long LQ, Li K (2012). MicroRNA-148a promotes myogenic differentiation by targeting the ROCK1 gene. J Biol Chem.

[13] Chen JF, Mandel EM, Thomson JM, Wu Q, Callis TE, Hammond SM, Conlon FL, Wang DZ (2006). The role of microRNA-1 and microRNA-133 in skeletal muscle proliferation and differentiation. Nat Genet.

[14] Chen JF, Tao Y, Li J, Deng Z, Yan Z, Xiao X, Wang DZ (2010). microRNA-1 and microRNA-206 regulate skeletal muscle satellite cell proliferation and differentiation by repressing Pax7. J Cell Biol.

[15] Goljanek-Whysall K, Pais H, Rathjen T, Sweetman D, Dalmay T, Munsterberg A (2012). Regulation of multiple target genes by miR-1 and miR-206 is pivotal for C2C12 myoblast differentiation. J Cell Sci.

[16] Zhang YQ, Li M, Wang H, Fisher W, Lin P, Yao QZ, Chen CY (2009). Profiling of 95 microRNAs in pancreatic cancer cell lines and surgical specimens by real-time PCR analysis. World J Surg.

[17] Huang Z, Huang S, Wang Q, Liang L, Ni S, Wang L, Sheng W, He X, Du X (2011). MicroRNA-95 promotes cell proliferation and targets sorting Nexin 1 in human colorectal carcinoma. Cancer Res.

[18] Huang X, Taeb S, Jahangiri S, Emmenegger U, Tran E, Bruce J, Mesci A, Korpela E, Vesprini D, Wong CS, Bristow RG, Liu FF, Liu SK (2013). miRNA-95 mediates radioresistance in tumors by targeting the sphingolipid phosphatase SGPP1. Cancer Res.

[19] Chen XC, Chen SM, Hang WJ, Huang HT, Ma HT (2014). MiR-95 induces proliferation and chemo- or radioresistance through directly targeting sorting nexin1 (SNX1) in non-small cell lung cancer. Biomed Pharmacother.

[20] Srinivas G, Kusumakumary P, Nair MK, Panicker KR, Pillai MR (2000). Mutant p53 protein, Bcl-2/Bax ratios and apoptosis in paediatric acute lymphoblastic leukaemia. J Cancer Res Clin Oncol.

[21] Nicolaides NC, Kinzler KW, Vogelstein B (1995). Analysis of the 5' region of PMS2 reveals heterogeneous transcripts and a novel overlapping gene. Genomics.

[22] Kim JY, Kang YS, Lee JW, Kim HJ, Ahn YH, Park H, Ko YG, Kim S (2002). P38 is essential for the assembly and stability of macromolecular tRNA synthetase complex: implications for its physiological significance. Proc Natl Acad Sci U S A.

[23] Kim MJ, Park BJ, Kang YS, Kim HJ, Park JH, Kang JW, Lee SW, Han JM, Lee HW, Kim S (2003). Downregulation of FUSE-binding protein and c-myc by tRNA synthetase cofactor p38 is required for lung cell differentiation. Nat Genet.

[24] Han JM, Park BJ, Park SG, Oh YS, Choi SJ, Lee SW, Hwang SK, Chang SH, Cho MH, Kim S (2008). AIMP2/p38, the scaffold for the multi-tRNA synthetase complex, responds to genotoxic stresses via p53. Proc Natl Acad Sci U S A.

[25] Kim S (2003). Genetic disruption of p38 / JTV1 in mice unveiled its pleiotropic activity as a novel tumor suppressor. DBPIA.

[26] Dey BK, Gagan J, Dutta A (2011). miR-206 and -486 induce myoblast differentiation by downregulating Pax7. Mol Cell Biol.

[27] Charge SB, Rudnicki MA (2004). Cellular and molecular regulation of muscle regeneration. Physiol Rev.

[28] Wigmore PM, Stickland NC (1983). Muscle development in large and small pig fetuses. J Anat.

[29] Sabourin LA, Rudnicki MA (2000). The molecular regulation of myogenesis. Clin Genet.

[30] Baskin KK, Winders BR, Olson EN (2015). Muscle as a “mediator” of systemic metabolism. Cell Metab.

[31] Ojima K, Ichimura E, Yasukawa Y, Wakamatsu J, Nishimura T (2015). Dynamics of myosin replacement in skeletal muscle cells. Am J Physiol Cell Physiol.

[32] Sarkar S, Dey BK, Dutta A (2010). MiR-322/424 and -503 are induced during muscle differentiation and promote cell cycle quiescence and differentiation by down-regulation of Cdc25A. Mol Biol Cell.

[33] Guo SL, Peng Z, Yang X, Fan KJ, Ye H, Li ZH, Wang Y, Xu XL, Li J, Wang YL, Teng Y, Yang X (2011). miR-148a promoted cell proliferation by targeting p27 in gastric cancer cells. Int J Biol Sci.

[34] Takagi S, Nakajima M, Mohri T, Yokoi T (2008). Post-transcriptional regulation of human pregnane X receptor by micro-RNA affects the expression of cytochrome P450 3A4. J Biol Chem.

[35] Huang Y, Zou Q, Song H, Song F, Wang L, Zhang G, Shen X (2010). A study of miRNAs targets prediction and experimental validation. Protein Cell.

[36] Miyake M, Hayashi S, Taketa Y, Iwasaki S, Watanabe K, Ohwada S, Aso H, Yamaguchi T (2010). Myostatin down-regulates the IGF-2 expression via ALK-Smad signaling during myogenesis in cattle. Anim Sci J.

[37] Allen DL, Du M (2008). Comparative functional analysis of the cow and mouse myostatin genes reveals novel regulatory elements in their upstream promoter regions. Comp Biochem Physiol B Biochem Mol Biol.

[38] Allen DL, Unterman TG (2007). Regulation of myostatin expression and myoblast differentiation by FoxO and SMAD transcription factors. Am J Physiol Cell Physiol.

[39] McPherron AC, Huynh TV, Lee SJ (2009). Redundancy of myostatin and growth/differentiation factor 11 function. BMC Dev Biol.

[40] Yang W, Zhang Y, Li YF, Wu ZG, Zhu DH (2007). Myostatin induces cyclin D1 degradation to cause cell cycle arrest through a phosphatidylinositol 3-kinase/AKT/GSK-3 beta pathway and is antagonized by insulin-like growth factor 1. J Biol Chem.

[41] Lennert K, Kaiserling E, Mazzanti T (1978). Diagnosis and differential diagnosis of lymphoepithelial carcinoma in lymph nodes: histological, cytological and electron-microscopic findings. IARC Sci Publ.

[42] Cai C, Qian L, Jiang S, Sun Y, Wang Q, Ma D, Xiao G, Li B, Xie S, Gao T, Chen Y, Liu J, An X (2017). Loss-of-function myostatin mutation increases insulin sensitivity and browning of white fat in Meishan pigs. Oncotarget.

[43] Hitachi K, Nakatani M, Tsuchida K (2014). Myostatin signaling regulates Akt activity via the regulation of miR-486 expression. Int J Biochem Cell Biol.

