# Supplementary material for: MicroRNA-95 promotes myogenic differentiation by down-regulation of aminoacyl-tRNA synthase complex-interacting multifunctional protein 2
Source: Oncotarget. 2017 Nov 30;8(67):111356–68. doi: 10.18632/oncotarget.22796 (PMC5762327; doi:10.18632/oncotarget.22796)
Supplement: Supplementary file 1 [file oncotarget-08-111356-s001.pdf]

MicroRNA-95 promotes myogenic differentiation by down-regulation of aminoacyl-tRNA synthase complex-interacting multifunctional protein 2

SUPPLEMENTARY MATERIALS

Supplementary Table 1: Primers for real-time PCR

Gene name	mRNA accession number	Sequence (5'→3')	Amplicon length (bp)
MHC	NM_010855	F: GGAGGACCAAGTGAGTGAGC	250
		R: TTTCGTCTAGCTGGCGTGAG	
Myogenin	NM_031189	F: ATCCAGTACATTGAGCGCCT	242
		R: TCCACGATGGACGTAAGGGA	
Aimp2	NM_001172146	F: AACCTGCATGTACCGGCTC	104
		R: CAAGGGCTTGCAAAGAAGGC	
U6	NR_004394	F: GCTTCGGCAGCACATATACTAAAAT	89
		R: CGCTTCACGAATTTGCGTGTCAT	
